# A randomised controlled trial of multiple periods of outdoor free-play to increase moderate-to-vigorous physical activity among 3 to 6 year old children attending childcare: study protocol

**DOI:** 10.1186/s12889-016-3604-x

**Published:** 2016-09-02

**Authors:** Luke Wolfenden, John Wiggers, Philip Morgan, Lubna Abdul Razak, Jannah Jones, Meghan Finch, Rachel Sutherland, Christophe Lecathelinais, Karen Gillham, Sze Lin Yoong

**Affiliations:** 1 Hunter New England Population Health, Locked Bag 10, Wallsend, NSW 2287 Australia; 2 School of Medicine and Public Health, University of Newcastle, Callaghan, NSW 2308 Australia; 3 Hunter Medical Research Institute, Newcastle, NSW 2300 Australia; 4 Priority Research Centre for Health Behaviour, University of Newcastle, Callaghan, NSW 2308 Australia; 5 School of Education, Priority Research Centre for Physical Activity and Nutrition, University of Newcastle, Callaghan, NSW 2308 Australia

**Keywords:** Physical activity, Randomised controlled trial, Intervention, Childcare, Preschool, Outdoor play, Activity breaks

## Abstract

**Background:**

The implementation of physical activity interventions in centre-based childcare services has been recommended to improve child health. This study aims to evaluate the efficacy of scheduling multiple periods of outdoor free play in increasing the time children spend in moderate-to-vigorous physical activity (MVPA) during childcare.

**Methods:**

The study will employ a between group cluster randomised controlled trial design. Fourteen childcare services in the Hunter New England region of New South Wales, Australia, who currently implement a single session of free outdoor play between their core operational hours of 9 am to 3 pm will be recruited into the trial. Childcare services will be randomised to an intervention or a no intervention control group. Childcare services in the intervention group will be supported by an early childhood education specialist to provide three periods of outdoor free play for children between the hours of 9 am to 3 pm. Each period of outdoor free play will be at least 15 min in duration but must equate to their total usual duration of outdoor play. Services in the control group will continue to implement a single period of outdoor play. The primary trial outcome is minutes of time children spend in MVPA whilst in care assessed objectively via accelerometer over 5 days. Outcome assessment will occur at baseline and 3 months post baseline. Generalised Linear Mixed Models (GLMM) under an intention to treat framework will be used to compare differences between groups in the primary trial outcome at follow-up. Sensitivity analysis will be conducted to test assumptions of missing data. Per protocol analysis will be performed using services that implemented the intervention as intended and subgroup analysis undertaken by gender and baseline physical activity levels of children.

**Discussion:**

The study tests a simple ecological intervention that has the potential to increase child physical activity in care.

**Trial registration:**

Australian New Zealand Clinical Trials Registry 12616000347460. Prospectively registered 17th March 2016.

## Background

Inadequate physical activity is associated with the most prevalent causes of mortality and morbidity including obesity, diabetes, cardiovascular disease and some cancers [1]. Physical activity in early childhood has immediate beneficial effects on blood pressure, lipid profile, motor skill and bone development [2–4] with greatest benefit accruing at moderate-to-vigorous intensity [3, 4]. Despite these benefits, research in Australia and internationally has demonstrated that most children aged 2 to 6 years do not engage in physical activity consistent with current national guidelines [5, 6].

A number of characteristics of centre-based childcare services suggest that they represent an ideal setting for interventions to improve physical activity in young children. First, centre-based childcare provides access to a significant proportion of the population aged less than 5 years, often for prolonged periods. For example, approximately 55 % of Australian children aged 0 to 5 years attended some form of centre-based care in 2014 [7]. Second, reviews suggest that young children are not sufficiently active during attendance at centre-based care, necessitating interventions in this setting [8]. Third, childcare service staff believe in the importance of children being physically active as a part of their professional responsibility and are amenable to interventions to support improvements in child activity [9].

Modifying the frequency of outdoor free-play may represent a potentially innovative and effective strategy in improving the physical activity of children attending childcare. Unstructured outdoor free-play (as opposed to structured, staff-guided play) has been consistently associated with greater child physical activity among children in care [10, 11]. However, findings of a randomised trial suggest that increasing the duration of time that children in childcare have available for outdoor free-play may not be effective in improving child physical activity [12]. A likely explanation for this is that extending outdoor free-play time alone does not account for children’s natural physical activity patterns. Children’s activity in care is characterised by short, intense bouts of activity of between 3 and 15 min occurring at the start of outdoor free-play opportunities, followed by extended recovery periods of sedentary behaviour or light activity [13–16]. As such, extending periods of outdoor play alone may not increase child moderate-to-vigorous physical activity (MVPA). However, even if the total duration of outdoor free-play time for children remains constant, scheduling shorter but more frequent opportunities for outdoor free-play may enhance child physical activity by promoting more spontaneous bouts of intense activity (‘activity peaks’) that is characteristic of the first 15 min of outdoor free-play [13].

In this context the aim of this study is to assess the efficacy of scheduling multiple periods of outdoor free-play per day in increasing the time children spend in MVPA during childcare relative to one outdoor play time of the same duration. This manuscript describes the trial methods and trial outcomes of the study.

## Methods

### Design

**Fig. 1:**
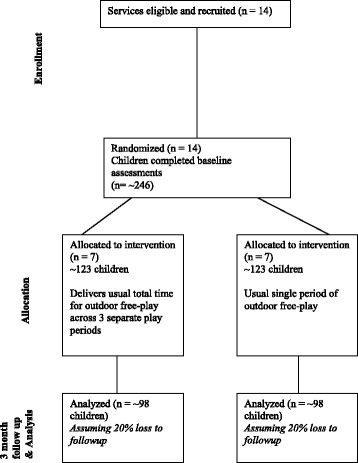
Flow of participants through each stage

The study will employ a between group, cluster randomised controlled trial design (see Fig. 1). Fourteen centre-based childcare services with one period of outdoor free-play (of at least 45 min duration) will be randomised to an intervention or control group. Intervention services will change their scheduling of outdoor free-play such that their usual total time for outdoor free-play is broken into multiple separate periods of at least 15 min in duration each with an indoor period of at least 30 min in between. Control services will continue to provide their usual total period of outdoor free-play time across a single continuous period. Intervention efficacy will be determined by comparing differences between groups in the minutes children spend in MVPA per day at childcare. MVPA will be assessed via accelerometer over 5 days at baseline and 3 months later. Approval to conduct the study was obtained from Hunter New England Human Research Ethics Committee (reference number 15/11/18/4.03) and the University of Newcastle Human Research Ethics Committee (reference number H-2016-0088).

### Participant eligibility and evidence-based recruitment strategy

#### Childcare services

To be eligible to participate in the trial, centre-based childcare services (defined as long day care services or preschools) will be required to have an enrolment of least 25 children aged between 3 to 6 years, be located within the Hunter or New England regions of New South Wales, Australia, and have only one outdoor free-play session occurring between the core operational hours of 9 am to 3 pm. Childcare services catering solely for special needs populations, or those participating in other physical activity interventions will be excluded from participating in the trial.

A list of all childcare services that are licensed to provide care for 3 to 6 year-old children located within the study region will be obtained from the licensing agency. Supervisors of childcare services across the study region will be sent study information prior to telephone contact to assess eligibility and to invite study participation among eligible services. Recruitment will continue until 14 services have consented to participate. Previous studies in childcare utilising this recruitment approach have yielded a childcare service participation rate of 81–84 % [17–19].

#### Children

Active parental consent will be required for study participation. To be eligible for the data collection component of the study, children will be required to be aged 3 to 6 years and attend participating services between 9 am and 3 pm on one or more days in the week of data collection. Children with an intellectual or physical impairment that may impact on their physical activity capacity or prevent them from complying with data collection protocols will be excluded.

To maximise study participation, children will be recruited utilising an evidenced-based strategy recommended for obtaining active parental consent for child participation in school-based research, which will include a mail out of study information and consent forms prior to onsite recruitment, face to face dissemination of information sheets and provision of consent forms to parents during periods of drop-off or pick-up from childcare [20]. Such a recruitment strategy has been utilised in previous trials of interventions for preschool aged children in this setting [21–23].

### Randomisation, allocation and blinding

A statistician with no other involvement in recruitment or data collection will allocate services to either the intervention or control condition in a 1:1 ratio using a computerised random number generator. Randomisation of childcare services will be stratified by the socioeconomic status of the area where the service is located, and on the service type (long day care service or preschool) based on evidence of an association between these factors and the physical activity policies and practices of services [24].

### Intervention

The intervention will seek to create a childcare environment more supportive of child physical activity by scheduling multiple opportunities for outdoor free-play in a way which is consistent with children’s developing fitness levels [25] and natural physical activity patterns [10, 13, 26]. Specifically, within a 6 h day (9 am to 3 pm) the intervention will divide their usual total time of outdoor free-play for children across three periods of at least 15 min duration.

Three periods of outdoor free-play was selected as: i) research has demonstrated repeated spikes in activity across the day in childcare coinciding with scheduled breaks [13, 16]; and ii) that the start of such breaks stimulate between 3 and 15 min of intense activity, sufficient to achieve an additional 10 min MVPA [27]. The minimum period of outdoor free-play of 15 min was selected given evidence that MVPA often attenuates after this period [13]. Furthermore, services will be required to schedule at least 30 min of indoor time (structured or unstructured play) between periods of outdoor free-play, based on evidence that children may be more likely to be moderate-to-vigorously physically active following prolonged indoor periods [28]. Immediately following baseline data collection, intervention services will be supported by a member of the research team and an early childhood education specialist to implement the intervention. This support will include written materials and at least two site visits and two telephone support calls prior to follow-up data collection to re-orient their operations to incorporate the scheduling change. No other intervention support will be provided to children, childcare service staff or parents.

### Control

Services allocated to the control group will schedule their usual single period of outdoor free-play for children across the day. Control services will also be instructed to continue the one outdoor free-play period of the same duration (except during inclement weather) across the study period.

### Data collection and measures

Data will be collected at baseline and approximately 3 months post baseline.

#### Primary trial outcome—minutes spent in moderate-to-vigorous physical activity in care

The primary trial outcome is the number of minutes children spend in MVPA during the core hours of service operation (9 am to 3 pm). MVPA will be objectively assessed using an Actigraph GT3X+ accelerometer using recommended cut-points [29]. The Actigraph accelerometer has established utility, validity and reliability and is the current gold standard for assessment of activity in children aged 3 to 6 years old [30].

Accelerometers will be worn by children during the core operational hours of childcare (9 am to 3 pm). Two data collectors, not blinded to allocation at follow-up, will attend services to fit and collect accelerometers using a standard protocol [29]. Accelerometers will be placed above the iliac crest at the hip of the child using a clip or band. Accelerometer data will be collected on every day of 1 week (5 days in total) of the data collection period at baseline and follow-up. Children will wear the accelerometer each day (up to 5 days) that they attend care. The accelerometer will be fitted as the children arrive at the childcare service and removed at 3 pm or earlier when the child departs the service. Despite evidence to suggest that increases in child activity at childcare do not result in decreased activity at home [31], at baseline and follow-up, consent will be sought for children to wear accelerometers during ‘out of care’ hours also to assess potential compensatory effects.

#### Secondary trial outcomes

Secondary trial outcomes include total child activity (counts per minute collected in 5 s epochs) in care [32] and percent of time children spend in MVPA adjusted for wear time, assessed via accelerometer. Additionally, as a potential adverse effect of the intervention, during interviews with childcare services, the number of injuries requiring documentation will be assessed using items taken from previous childcare physical activity studies [21].

#### Child and parent characteristics

A computer-assisted telephone interview with parents will be conducted to collect: child and parent demographic information (age, gender, household income, and parent education); usual parent physical activity; and child and parent height and weight, using items from the New South Wales Population Health Survey [33] and items to assess the home environment from the preschool age physical activity questionnaire (Pre-PAQ) questionnaire [34]. The survey will be conducted at baseline and follow-up.

#### Service characteristics

During a telephone interview with supervisors of participating childcare services the following service characteristics will be assessed: postcode of locality (to assess the socioeconomic status of the area) [35]; number of years in operation; total number of 3 to 6 year-old children enrolled; number of staff; staff qualifications; and service governance (Department of Education service or privately owned). Such items were drawn from previous studies [17, 24].

#### Service outdoor free-play schedule and physical activity environment

Observations at childcare services will be conducted by data collectors (not blind to group allocation) to record the duration (via stop watch), timing and frequency of outdoor free-play to ensure that services are implementing outdoor free-play periods consistent with the study protocol. Data collectors will also collect information regarding the childcare service physical activity environment using a comprehensive environment assessment tool (Environment and Policy Assessment and Observation instrument, EPAO) [36]. The following types of physical activity observation elements will be conducted: Active play opportunities, sedentary opportunities, sedentary environment, portable play environment, fixed play environment, staff behaviour physical activity, physical activity training and education and physical activity policy.

### Intervention fidelity

The research team will visit the services during the intervention period to observe if childcare services have implemented the intervention prior to follow-up data collection. A checklist developed by the research team will be used to monitor whether the intervention was delivered as per the study protocol on the day of data collection.

### Analysis

Minutes of MVPA will be determined using age-specific child-validated equations (cut points) [29]. Generalised Linear Mixed Models (GLMM), to take account of the repeated measures on children (daily measures during baseline and follow-up periods) as well as clustering of individuals within services, will be used under an intention to treat framework to test for a difference in change in minutes of MVPA between groups. The GLMM will include terms for time (baseline and follow-up), group (intervention or control group), and the interaction of time and group, and will control for child gender and total outdoor play time. A sensitivity analysis will be performed using multiple imputation for missing data to assess robustness of the main analysis [37]. Per protocol analysis will be performed using services that implemented the intervention as intended and subgroup analysis undertaken by gender and baseline physical activity levels of children.

### Sample size and power calculation

The study will approach approximately 500 children from 14 childcare services across the study region. Assuming the standard deviation of MVPA is 2.7 min/h [38] and assuming an intraclass correlation coefficient of 0.1 [39], a sample of 14 children per cluster (assuming a conservative participation rate of approximately 50 and a 20 % loss to follow-up) will provide the study with 80 % power to detect a change of 9.9 min in MVPA. An increase of 10 min of MVPA in children aged 3 to 6 years old has been found to have clinically significant beneficial effects on fat mass and peak bone mass [3, 4].

## Discussion

Supporting physical activity in early childhood is a recommended strategy [40] to reduce the community health burden of inactivity, as physical activity in childhood persists over time [41], and health behaviours in childhood are more easily influenced than behaviours in adolescents and adults [42]. While previous observational studies have reported that children are frequently sedentary or engaged in light activity, in recent years has research began to accumulate that describes patterns of activity among children aged 3 to 6 years old across a day in childcare [10, 13, 16]. Through the use of experimental methods, the trial will provide rigorous evidence to indicate whether interventions sensitive to child activity patterns by increasing the frequency of bouts of outdoor free-play are effective. Even if moderately effective, the intervention has the potential to improve the health and wellbeing of the hundreds of thousands of Australian children who attend some form of centre-based childcare through reducing the risk of the precursors of chronic disease.
